# Circulatory Astrocyte and Neuronal EVs as Potential Biomarkers of Neurological Dysfunction in HIV-Infected Subjects and Alcohol/Tobacco Users

**DOI:** 10.3390/diagnostics10060349

**Published:** 2020-05-28

**Authors:** Sunitha Kodidela, Kelli Gerth, Namita Sinha, Asit Kumar, Prashant Kumar, Santosh Kumar

**Affiliations:** 1 College of Pharmacy, Department of Pharmaceutical Sciences, University of Tennessee Health Science Center, 881 Madison Ave, Memphis, TN 38163, USA; skodidel@uthsc.edu (S.K.); kgerth1@uthsc.edu (K.G.); nsinha2@uthsc.edu (N.S.); akumar23@uthsc.edu (A.K.); 2 Department of Pediatrics, University of Tennessee Health Science Center and Le Bonheur Children’s Hospital, Memphis, TN 38103, USA; pkumar21@uthsc.edu

**Keywords:** HIV, plasma extracellular vesicles (EVs)/exosomes, alcohol, tobacco, GFAP, L1CAM, neurocognition

## Abstract

The diagnosis of neurocognitive disorders associated with HIV infection, alcohol, and tobacco using CSF or neuroimaging are invasive or expensive methods, respectively. Therefore, extracellular vesicles (EVs) can serve as reliable noninvasive markers due to their bidirectional transport of cargo from the brain to the systemic circulation. Hence, our objective was to investigate the expression of astrocytic (GFAP) and neuronal (L1CAM) specific proteins in EVs circulated in the plasma of HIV subjects, with and without a history of alcohol consumption and tobacco smoking. The protein expression of GFAP (*p* < 0.01) was significantly enhanced in plasma EVs obtained from HIV-positive subjects and alcohol users compared to healthy subjects, suggesting enhanced activation of astrocytes in those subjects. The L1CAM expression was found to be significantly elevated in cigarette smokers (*p* < 0.05). However, its expression was not found to be significant in HIV subjects and alcohol users. Both GFAP and L1CAM levels were not further elevated in HIV-positive alcohol or tobacco users compared to HIV-positive nonsubstance users. Taken together, our data demonstrate that the astrocytic and neuronal-specific markers (GFAP and L1CAM) can be packaged in EVs and circulate in plasma, which is further elevated in the presence of HIV infection, alcohol, and/or tobacco. Thus, the astroglial marker GFAP and neuronal marker L1CAM may represent potential biomarkers targeting neurological dysfunction upon HIV infection and/or alcohol/tobacco consumption.

## 1. Introduction

Chronic HIV infection can lead to CNS complications, in addition to affecting the peripheral immune system [1,2]. With the introduction of antiretroviral therapy (ART), peripheral complications have dramatically reduced [3]. However, the CNS complications tend to progress more rapidly due to inadequate levels of ART in the brain, the presence of a latent virus, and persistent viral replication [4,5]. This is supported by evidence that viral RNA was detectable in the CSF of HIV-infected subjects who had complete suppression of plasma viral load with ART [6,7]. Persistent viral replication in the CNS can lead to neuroinflammation, neurodegeneration, and subsequently, neurocognitive disorders [8,9]. HIV-associated neurocognitive disorders (HAND) affect almost 30%–60% of infected individuals, and they comprise clinical symptomatology ranging from asymptomatic to minor cognitive motor disorders [10,11], which are mainly prevalent in subjects who are not virally suppressed with ART [10,12]. These complications are further exacerbated in the presence of substance abuse [13,14]. Substance abuse, particularly alcohol consumption and tobacco smoking, is known to exacerbate HIV replication and its associated complications [15,16,17,18,19,20]. Given that these early pathological changes are asymptomatic, rather than relying on clinical manifestations, biomarkers are necessary for early identification and for follow up of future cases. 

Identifying biomarkers to diagnose neurocognitive dysfunctions using MRI and PET scan-based methods is expensive, and withdrawal of CSF for this purpose is an invasive and tedious method. Therefore, there is a need for noninvasive biomarkers to address the same pathologies. Plasma extracellular vesicles (EVs) can serve as reliable markers due to their ability to package and transport various biological molecules across the CNS. Several reports suggest a bidirectional transport of EVs from the brain to the systemic circulation via the blood–brain barrier (BBB) [21,22]. Further, plasma EVs can deliver their content to the CNS and can cause neuroinflammation [23]. Moreover, plasma EV content is altered under HIV, alcohol, and tobacco use [24,25,26]. EVs from neuronal and astrocytic cells can be circulated in plasma [27,28,29] and thus have the potential to serve as biomarkers to predict ongoing disease progression in the CNS. Hence, in this pilot study, our objective was to investigate the expression of astrocytic and neuronal-specific proteins in EVs circulated in the plasma of HIV subjects, with and without a history of alcohol consumption and tobacco smoking. We propose the hypothesis that the expression of neuronal and astrocytic markers associated with neurocognitive impairment/neuroinflammation is elevated in EVs of patients with HIV, and their expression is altered in the presence of alcohol and tobacco use. 

## 2. Materials and Methods

### 2.1. Study Population

A total of 23 previously recruited subjects from Cameroon, Africa, available from earlier studies [16], were used in the present study. In this study, subjects were assigned into 6 different groups: (a) four healthy subjects who were HIV-negative and reported to be nonsmokers/alcohol users (healthy); (b) four HIV-positive subjects without a history of alcohol consumption or tobacco smoking (HIV); (c) four HIV-negative alcohol drinkers (drinkers); (d) four HIV-negative tobacco smokers (smokers); (e) three HIV-positive alcohol drinkers (HIV+drinkers); (f) four HIV-positive tobacco smokers (HIV+smokers). All procedures performed in the present study involving human participants were in accordance with the Code of Ethics of the World Medical Association (Declaration of Helsinki). Informed consent was obtained from all individual participants included in the study. Subjects were recruited upon approval of the Institutional Review Board (IRB) from the University of Missouri-Kansas City (UMKC IRB# 10-84e, Dated 09/02/11) and the University of Tennessee Health Scienter (IRB# 16-04348XP, Dated 09/01/16), as well as from the IRB/Institutional Ethics Committee (IEC) from Provincial Regional Hospital, Ministry of Public Health, Bamenda, Cameroon (FWA #A00017110: Dated, 4/28/11) [16]. The age range of study subjects is 25–60 years. The inclusion criteria of the subjects were as follows: (1) HIV-positive subjects: CD4 of 200–500 cells/μL; (2) smokers: history of less than 20 pack years (a pack year: smoking at least one pack per day for one year); (3) alcohol drinkers: 7–14 drinks/week for male and 4–7 drinks/week for female. The exclusion criteria were: (1) infectious diseases, such as active TB, malaria, and hepatitis A/B/C; (2) individuals receiving ART or other drugs of abuse. 

### 2.2. Plasma EV Isolation and Characterization

We followed ISEV 2018 guidelines for the isolation, characterization, and validation of EVs [30]. Plasma was filtered using a 0.22-μm filter (Millipore) to remove particles greater than 0.2 μm in size. 100 uL of filtered plasma was processed through the Total Exosome Isolation Kit (Invitrogen), as per the manufacturer’s recommendation. We have optimized the precipitation-based method to isolate EVs from small volumes of clinical samples and validated the size of and shape of isolated EVs by JEOL 2000EXII transmission electron microscope (TEM) [31] (The Neuroscience Institute, University of Tennessee Health Science Center), as described previously [24,25]. Tunable resistive pulse sensing (TRPS) using a qNano gold instrument (Izon Science, Medford, MA) has been utilized to measure the size distribution and concentration of EVs [32]. To measure the zeta potential, the isolated EVs were subjected to dynamic light scattering using a Zetasizer Nano-ZS (Malvern Instruments Inc, Malvern, UK) [33]. We further validated the samples for expression of EV marker proteins CD63, CD81, and CD9 with Western blotting, by loading equal amounts of protein. Data on size distribution and concentration of EVs were attained using Izon Control Suite (v3.3 software, Medford, MA 02155, USA) according to the manufacturer’s instructions.

### 2.3. Western Blotting

The protein concentrations of EV samples were quantified using the Pierce BCA Protein Assay Kit (Thermo Scientific). Then, 25 µg of protein from each sample was loaded onto 10% sodium dodecyl sulfate-polyacrylamide acrylamide gel and the rest of the procedure was performed as described previously [19]. We evaluated the expression of protein markers associated with neuronal (L1 Cell Adhesion Molecule) and astrocytic (Glial fibrillary acidic protein) cells. The following primary antibodies, including GFAP (Polyclonal Rabbit Anti-Glial Fibrillary Acidic Protein, Dako Omnis, Catalog No. 2023-11), and L1CAM (Rabbit Polyclonal, Catalog No. 20659-1-AP, Proteintech Inc, Rosemont, IL, USA) were used. The dilution for all primary antibodies was 1:500. The goat anti-mouse and goat anti-rabbit secondary antibodies (LI-COR Biosciences) were utilized at a 1:10,000 dilution. The membranes were scanned on the LI-COR Biosciences Odyssey Sa Infrared Imaging System. Densitometry analyses of the proteins were performed using LI-COR Image Studio Software (v.5.2, Nebraska, USA)

### 2.4. Statistical Analysis

Statistical analyses were performed using GraphPad Prism software v. 5 (GraphPad Software Inc., San Diego, CA, USA). The relative protein expression levels were presented as mean ± SEM. The nonparametric *t*-test was used to compare the protein levels between the two groups. One-way ANOVA was used to compare three or more groups. *p* < 0.05 is considered significant and represented as “*”.

## 3. Results and Discussion

**Figure 1:**
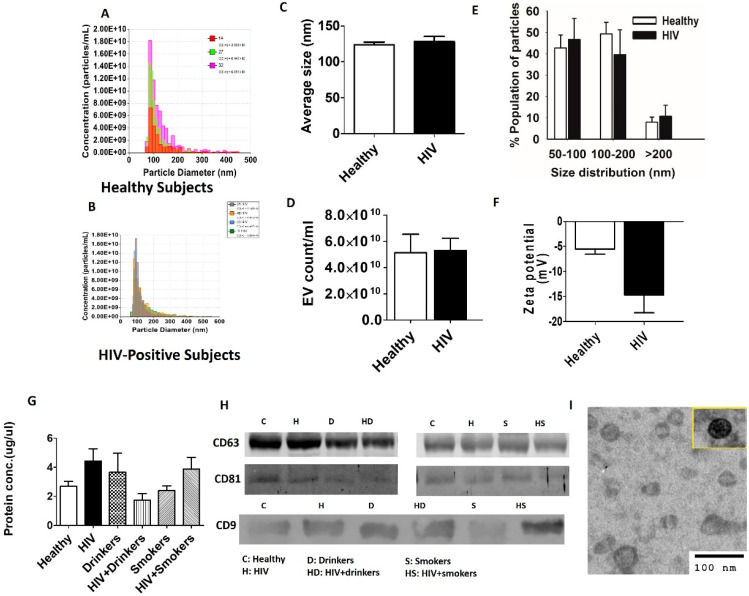
Characterization of plasma EVs. (**A**–**E**) Comparison of average concentration, size, and size distribution of isolated EVs from healthy HIV-positive subjects obtained using qNano. (**F**) Comparison of the average zeta potential of EVs isolated from healthy with HIV-positive subjects. (**G**) Comparison of total EV protein levels in different study groups. (**H**) Detection of exosomal marker proteins, CD63, CD81, and CD9 in different subjects from each study group by Western blotting. C—control, H—HIV, Dr—drinkers, HD—HIV+drinkers, S—smokers, HS—HIV+smokers. (**I**) Identification and validation of human plasma-derived EVs by transmission electron microscopy (TEM). All bars indicate mean ± SEM values. *p* < 0.05 is considered significant. Unpaired *t*-test was used to compare two groups. ANOVA was used to test the differences between multiple groups.

The isolated EVs from plasma samples of healthy and HIV-positive subjects were characterized for their size, zeta potential, and EV number (Figure 1A–E). The results did not show a significant difference in EV size and their relative size distributions, zeta potential, or EV concentrations between healthy and HIV-positive subjects (Figure 1A–E). We also measured the protein concentrations in EVs isolated from plasma samples of HIV, Drinkers, HIV + Drinkers, Smokers, and HIV + Smokers and compared them with the protein concentrations in EVs from healthy subjects (Figure 1F). Although there appears to be a slight increase in protein concentration from HIV subjects, in general, protein concentrations in the EVs did not vary significantly among study groups (Figure 1G). In addition, we verified the presence of the EV marker proteins CD63, CD81, and CD9 with Western blotting from each group (Figure 1H) by loading equal amounts of protein. The TEM of EVs isolated from healthy subjects (Figure 1I) showed a typical double-membraned structure of < 100 nm, suggesting the validity of the isolation method. 

**Figure 2:**
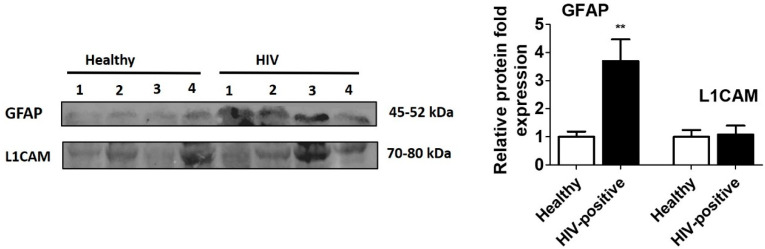
Expression of neuronal and astrocyte marker proteins in plasma EVs of Healthy and HIV-positive subjects. EVs were isolated from plasma of healthy (*n* = 4) and HIV-positive subjects (*n* = 4). Equal amounts of protein were loaded to analyze the expression of GFAP and L1CAM proteins in healthy as well as HIV-positive samples. GFAP expressions were found to be significantly high in HIV-positive subjects compared to healthy subjects, suggesting CNS damage in HIV-subjects. ** indicates *p* < 0.01, considered significant.

We investigated these plasma EVs to study the effects of HIV and substance use on CNS damage and associated neurological complications, as drawing blood is less expensive than neuroimaging and is minimally invasive compared to lumbar punctures required for CSF acquisition. For this, we evaluated the expression of GFAP and L1CAM proteins, which are reported to be EV markers of astrocytic and neuronal cells, respectively [34,35]. The protein expression of GFAP (*p* < 0.01) was significantly enhanced in plasma EVs obtained from HIV-positive subjects compared to healthy subjects (Figure 2; Supplementary Figure S1), suggesting enhanced activation of astrocytes due to HIV infection.

During neuroinflammation, when astrocytes are activated, they are characterized by an increase in size, number, and thickness of processes, as well as an increased level of GFAP expression [36]. GFAP expression is developmentally and pathophysiologically regulated. Elevated levels of GFAP are an important feature of the astrocytic reaction, which is frequently observed in brain damage or neurodegeneration [36,37,38] and in HIV-associated dementia [39]. Though we could not correlate the EV GFAP levels with neuropsychological impairment in our cohort due to lack of information, the subjects used in this study were likely to have a high probability of neuronal dysfunction. This speculation is based on the fact that the HIV-positive subjects were chronically infected and not on ART. Our results are supported by a study from Fan et al., where they reported that HIV-Tat treated astrocytes showed an upregulation of GFAP. This increased GFAP expression was associated with astrocyte-mediated Tat neurotoxicity [40]. Interestingly, GFAP levels in the CSF of HIV subjects with dementia were not significantly different from HIV subjects without dementia [41]. However, EVs derived from the CSF of HIV subjects with cognitive impairment had higher levels of GFAP compared to HIV subjects who did not have cognitive impairment [42]. This suggests a greater importance of EVs as a biomarker to predict HIV-associated neurological complications than CSF. Further, given the important role of EVs in cell–cell communication [43,44], packaging of GFAP protein in EVs and their circulation via plasma serve multiple purposes [28]. Plasma EVs not only serve as feasible and reliable biomarkers to predict HIV-associated neurological complications, but are also involved in physiological changes in peripheral organs upon delivering EV proteins to peripheral cells.

L1CAM plays an important role during neural development and in the regeneration of the adult nervous system [45]. Our results showed no significant increase in the level of L1CAM upon HIV infection compared to healthy subjects (Figure 2). Our finding is consistent with the finding from Guha et al., who also reported no difference in CSF EV L1CAM levels among patients with and without HAND [46], even though it was considered a specific marker for identifying neuron-derived EVs in the plasma of HIV, HAND, and Alzheimer’s disease subjects [27,29]. 

**Figure 3:**
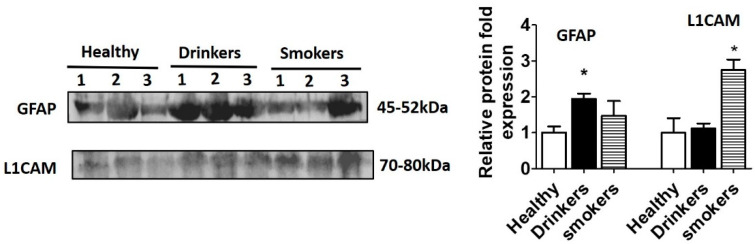
Expression of GFAP and L1CAM in plasma EVs of healthy subjects and substance users. EVs were isolated from plasma of Healthy (*n* = 3), alcohol Drinkers (*n* = 3) and cigarette smokers (*n* = 3). Equal amounts of protein were loaded to analyze the expression of GFAP and L1CAM proteins in these subjects. There was a significant difference in the expression of these proteins between healthy subjects and substance users. * indicates *p* < 0.05 is considered significant.

Alcohol use is prevalent among subjects with HIV infection. Moreover, chronic use of alcohol can lead to neuronal damage, inducing neurotoxicity through various mechanisms [47,48]. Therefore, we next studied the expression of GFAP and L1CAM in HIV-negative alcohol drinkers. Increased expression of GFAP in HIV-negative drinkers compared to healthy subjects suggests astrocytic activation in those subjects (Figure 3). 

Astrocytic activation in these drinkers probably suggests subsequent neuronal damage because astrocytes play a supportive role in neuronal functions, including neurotransmitter uptake [49] and the synthesis and secretion of neurotrophic factors [50]. GFAP has been found elevated in the plasma of patients with traumatic brain injury and was identified as a potential biomarker [51,52,53]. In addition, chronic exposure of alcohol induced GFAP immunoreactivity in astrocytes in the cerebral cortex [54], hippocampus [55], and in the cingulate cortex of adult rat brains [56]. 

In addition to alcohol, smoking is also known to be associated with neurotoxicity [57,58]. We observed elevated levels of L1CAM in HIV-negative smokers compared to healthy subjects, probably suggesting injured, apoptotic neuronal cells (Figure 3, Supplementary Figure S2). Our findings are supported by a study that showed increased levels of L1CAM in EVs derived from the plasma of smokers [59]. 

**Figure 4:**
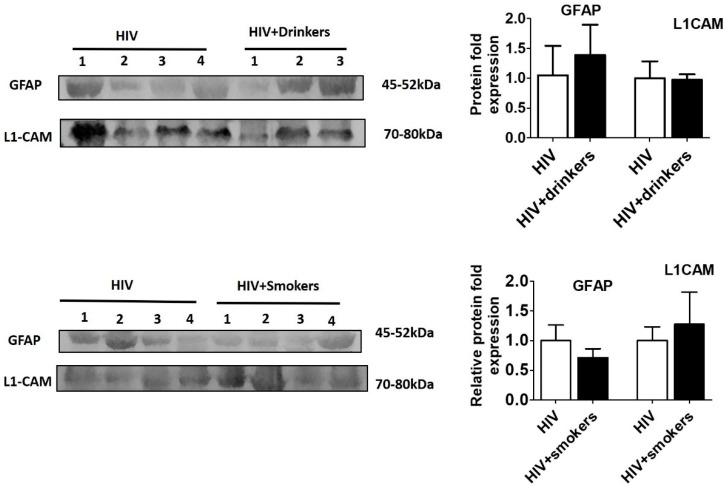
Expression of neuronal and astrocyte proteins in plasma EVs of HIV-positive subjects with and without substance use. EVs were isolated from plasma of HIV (*n* = 4), HIV-positive drinkers (*n* = 3), and HIV-smokers (*n* = 4). Equal amounts of EV protein were loaded to analyze the expression of GFAP and L1CAM proteins. There were no significant differences in the expression of the proteins between HIV and HIV-positive substance users. *p* < 0.05 is considered significant.

Next, we investigated whether alcohol consumption and smoking further exacerbate brain damage and affect the expression of these markers in HIV subjects. Therefore, we compared the astrocytic and neuronal markers between HIV-positive nondrinkers/smokers and HIV-positive drinkers/smokers (Figure 4, Supplementary Figure S3). However, no significant difference was found in astrocytic and neuronal markers between the HIV-positive subjects who also consume alcohol or smoke tobacco compared to HIV-positive subjects without substance use. 

This observation could be due to several reasons, including saturated levels of these neuronal markers in plasma EVs from alcohol drinkers and/or smokers and their inability to further increase in the presence of both HIV and/or substance use. Some limitations in this study are a small sample size and exclusion of subjects who are on ART drugs. In the USA, almost all HIV-infected individuals are on ART as soon as they are diagnosed. In our future project, in addition to recruiting larger cohorts who are on ART, we plan to further isolate subpopulations of glial and neuronal EVs from plasma EVs using specific antibodies. We will then explore their role in cell–cell communication and neuronal dysfunction.

## 4. Conclusions

We investigated the selected plasma EVs components as potential biomarkers for HIV and/or substance use associated with CNS damage and associated neurological complications. This technique has an advantage, as drawing blood is less expensive than neuroimaging and is minimally invasive compared to lumbar punctures required for CSF acquisition. The present study has established that the astrocytic and neuronal-specific markers (GFAP and L1CAM), which have the potential to play a role in neurological dysfunctions, can be packaged in EVs and circulated in plasma. The increased levels of GFAP in plasma EVs from HIV subjects can serve as a potential biomarker and reliable indicator of neurological dysfunction in those subjects. Further, elevated levels of GFAP in drinkers and L1CAM in smokers without HIV infection suggest a role of alcohol and smoking in inducing CNS complications. A future study would include validation of these potential EV biomarkers using a large cohort in different HIV populations who are also on ART drugs.

## Supplementary Materials

The following are available online at https://www.mdpi.com/2075-4418/10/6/349/s1, Figure S1: Original Western blots of expression of neuronal and astrocyte marker proteins in plasma EV of Healthy and HIV-positive subjects, Figure S2: Original Western blots of expression of GFAP and L1CAM in plasma EVs of healthy and substance users, Figure S3: Original Western blots of expression of neuronal and astrocyte proteins in plasma EVs of HIV-positive subjects with and without substance use.
