# Supplementary material for: Circulatory Astrocyte and Neuronal EVs as Potential Biomarkers of Neurological Dysfunction in HIV-Infected Subjects and Alcohol/Tobacco Users
Source: Diagnostics (Basel). 2020 May 28;10(6):349. doi: 10.3390/diagnostics10060349 (PMC7345258; doi:10.3390/diagnostics10060349)

Circulatory Astrocyte and Neuronal EVs as Potential Biomarkers of Neurological Dysfunction in HIV-Infected Subjects and Alcohol/Tobacco Users

Sunitha Kodidela [1], Kelli Gerth [1], Namita Sinha [1], Asit Kumar [1], Prashant Kumar [2] and Santosh Kumar *

1 College of Pharmacy, Department of Pharmaceutical sciences, University of Tennessee Health Science Center, 881 Madison Ave, Memphis, TN, 38163, USA; skodidel@uthsc.edu (S.K.); kgerth1@uthsc.edu (K.G.); nsinha2@uthsc.edu (N.S.); akumar23@uthsc.edu (A.K.)

2 Department of Pediatrics, University of Tennessee Health Science Center and Le Bonheur Children’s Hospital, Memphis, TN 38103, USA; pkumar21@uthsc.edu

*Correspondence: ksantosh@uthsc.edu; Tel.: 901-448-7157-1

Figure A1: Original Western blots of expression of neuronal and astrocyte marker proteins in plasma EV of Healthy and HIV-positive subjects.

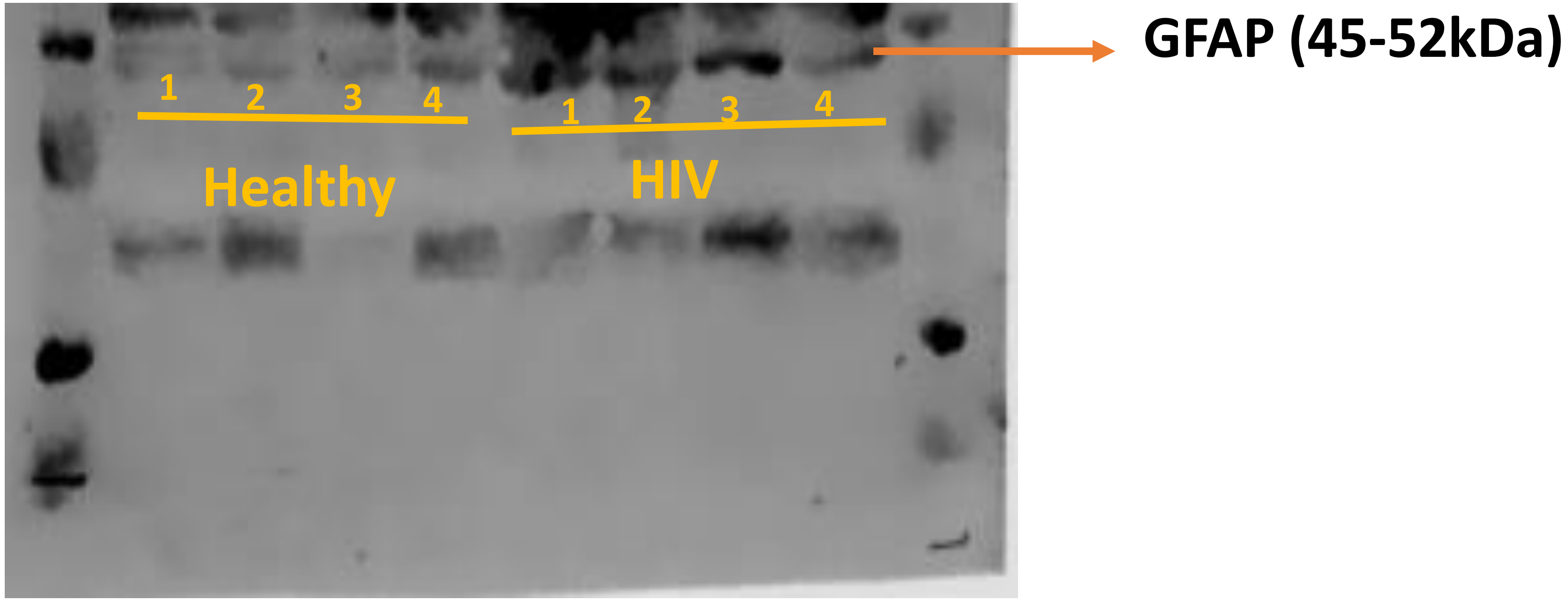

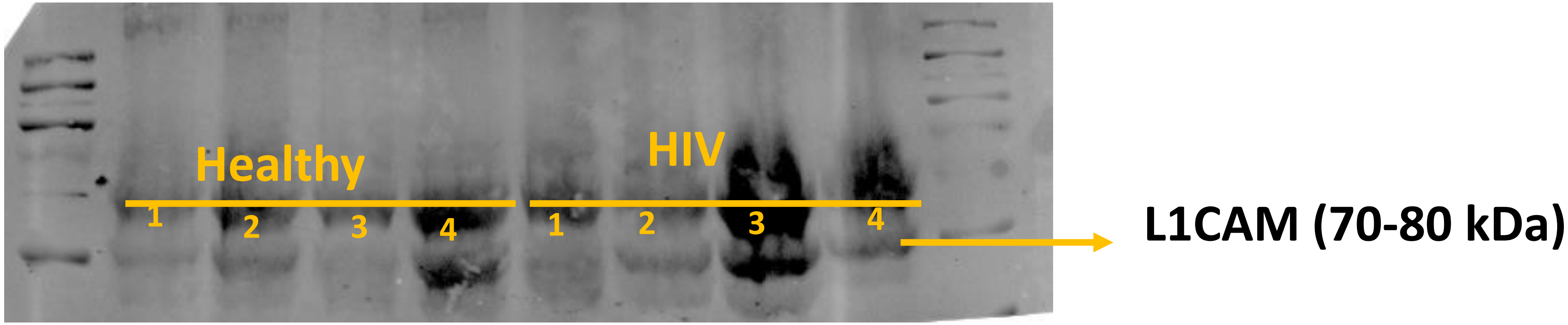

Figure A2: Original Western blots of expression of GFAP and L1CAM in plasma EVs of healthy and substance users.

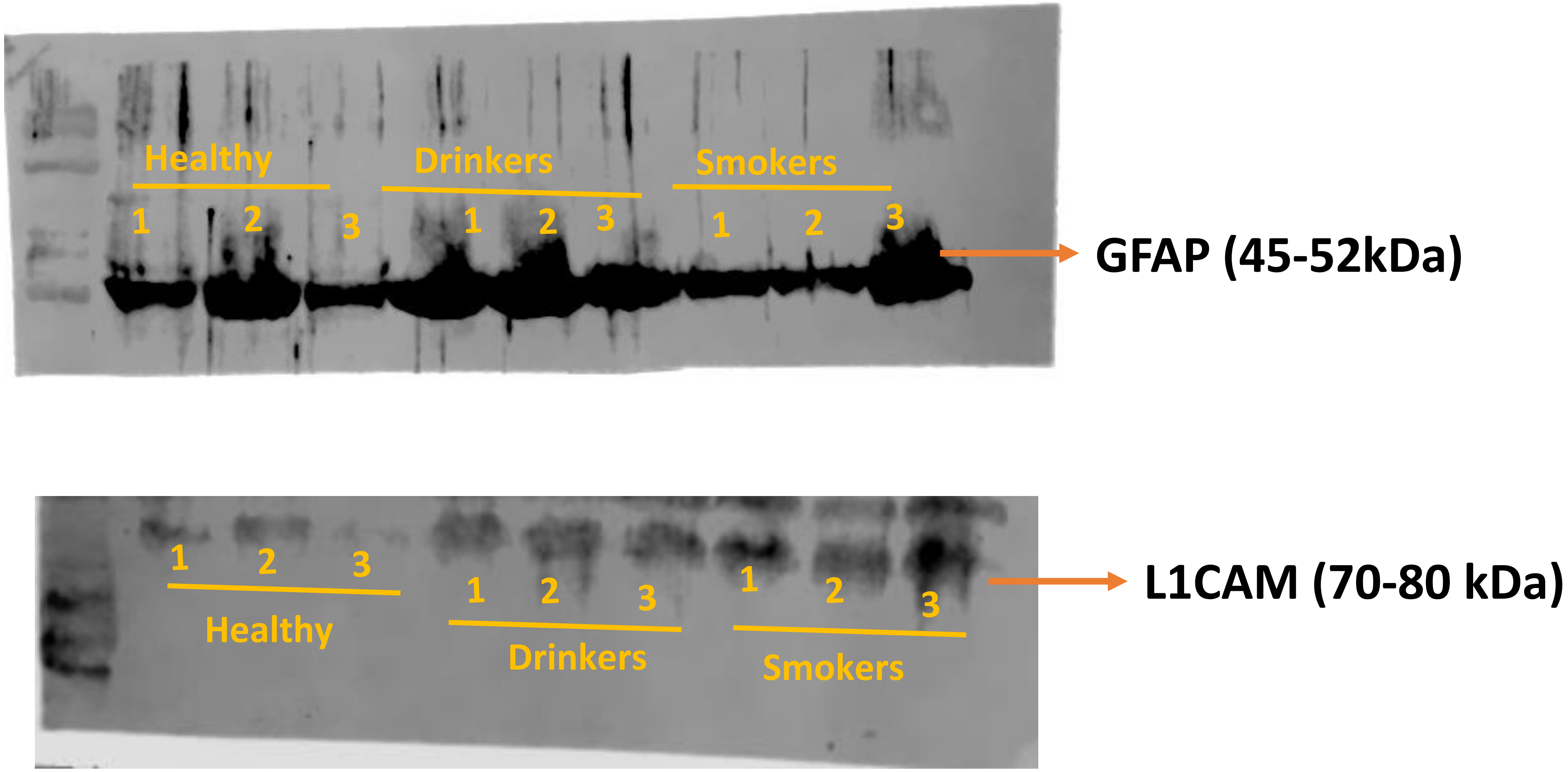

Figure A3: Original Western blots of expression of neuronal and astrocyte proteins in plasma EVs of HIV-positive subjects with and without substance use.

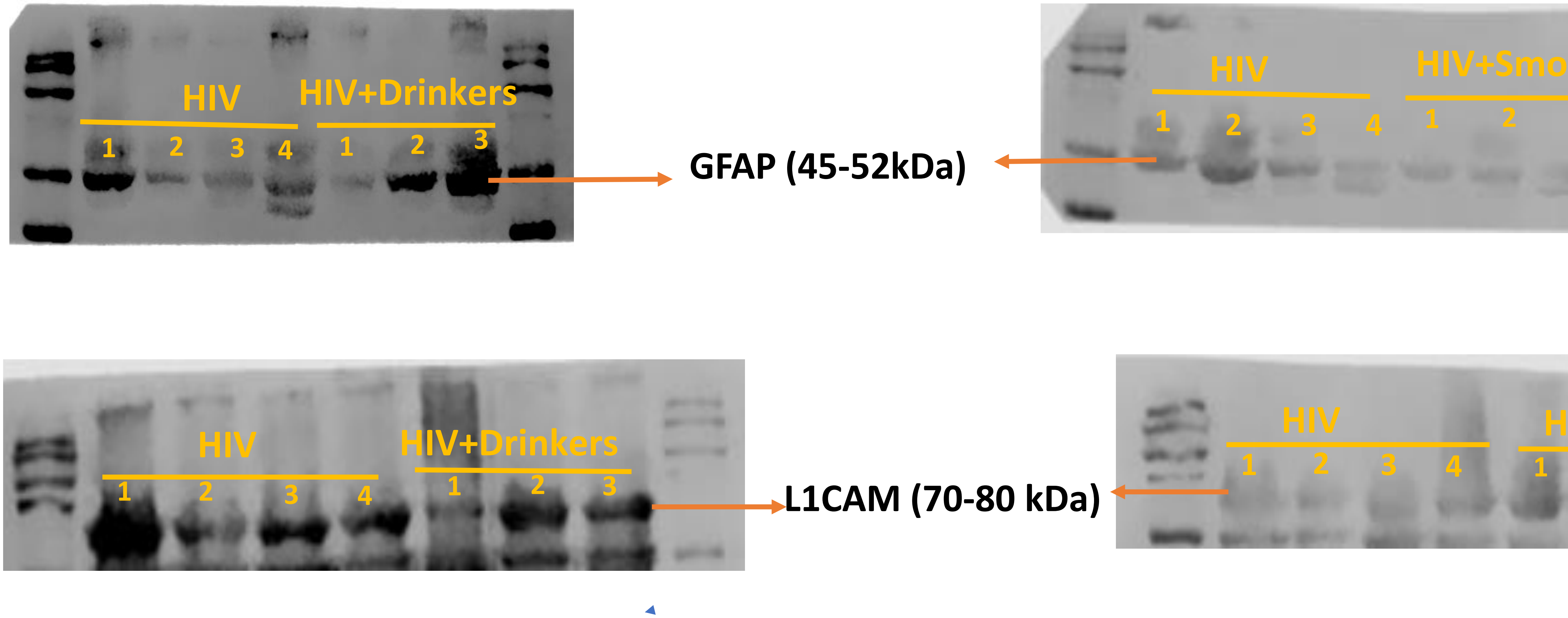

Supplement: Supplementary file 1 [file diagnostics-10-00349-s001.pdf]
